# Editorial Department

**Published:** 1885-01

**Authors:** 


### The Bistoury.

ELMIRA, N. Y., JANUARY, 1885.

The BISTOURY is published Quarterly, upon the first of April, July, October and January, at Fifty Cents a year in advance.

ONLY A CHRISTMAS CARD.—Some one, doubtless attracted by the drawing and coloring of the card, rather than by its truthfulness to nature, sends us a christmas card ornamented with a picture of a beautiful little girl seated on the ground, arranging a bunch of flowers, while five little kittens are frolicking at her feet.

That picture is not truthful. There never existed, nor will there ever appear, a little girl —unless she is a corpse—who will sit within reach of five playful kittens, and calmly arrange a boquet of flowers! Nonsense! Why, she would scatter the flowers to the winds, have a kitten under each arm, one in each hand, and try to gather in the fifth with her toes! That artist is a crusty old bachelor who never saw little girls at play five consecutive minutes in his life.

---

POISONOUS BEANS.—It is not generally known, nor is the fact stated in the U. S. Dispensatory, that the castor-oil bean is poisonous. The plant is extensively used for ornamenting gardens and grounds surrounding our dwellings. It bears large, fan-like leaves, a red stalk and several cluster of flowers, which, when ripe, are transformed into pods, containing the beans. These, the children are apt to appropriate by reason of their attractive appearance and occasionally have they been found tasting of them. Recently, we tried them to ascertain what sort of a savory morsel they were, and promptly was our curiosity satisfied in being made very sick thereby. Almost immediately dizziness ensued, [to the extent of staggering and falling had not we laid down] with extreme nauseau, accompanied with paralysis of the extremities. These symptoms lasted for nearly six hours, then, gradually disappeared. We had almost forgotten the circumstance until a month ago we were called in consultation with a physician to investigate the symptoms of poison, in a little girl aged perhaps, seven years. The attending physician decided she was suffering from some sort of poisoning that the symptons did not clearly reveal. She vomited constantly, had contraction of the muscles, particularly of the neck, drawing the head backward; enlarged abdomen, (*tympanities*); dilated pupils, and unconsciousness. She died the third day. Examination of the premises revealed that the children had been gathering castor beans from the withered plants in the front yard. Closer inspection of the beans exhibited that several of them had been nibbled, showing the imprints of the child's teeth. At last, it was definitely ascertained that the little one had eaten fragments of the beans, when the poisoning symptoms were fully accounted for.

Nowhere is the poisonous quality of the castor bean referred to We believe that very few people are familiar with its dangerous qualities. It should be banished from our yards and gardens as too perilous a plant to barbor.

---

THE HOSPITAL QUESTION.—"A descendant of Adam" writes to the *Daily Advertiser* of December 24th, calling the attention of "generous citizens and those interested in the monument to Adam," to the necessity for a new city hospital. She, (for the writer is a female descendant,) suggests that the "Home for the Aged" building might be secured for hospital purposes, enabling the managers of that institution to purchase a *smaller* and less expensive property, nearer the city.

As Cable's Ristofalo would say, "Yes, yes, that's all right, Mrs. Riley;" but, since the apartments of the aforementioned home are all occupied by old people, how are they to accommodate them in smaller quarters? Then, again, since by the terms of the conveyance of the property to the managers of the home for the aged by Dr. Eldridge, said property is to revert to his estate if ever used for any other purpose than that of a "home for the aged," how are the managers to sell it for a hospital?

That little game seems to be blocked—as it should be, since the location and building is well adapted to the wants of the old people, while it would be wholly inappropriate in both these particulars for the purpose suggested.

It is the hospital that needs to be "smaller and nearer the city"—not the home. The latter institution is a well managed, successful, and much needed charity, as its well filled wards, and comfortable and happy inmates fully attest. We hope that no attempt at curtailing its usefulness will ever be employed. As for the hospital, it is also needed, but any scheme to locate it outside the city limits, would seem to frustrate its usefulness for the sick and wounded for which it is proposed.

---

WHAT SAY THE COMMON COUNCIL ?—Some of our Fifth Ward and town of Southport citizens would like permission to drive faster than a walk over the bridges. They complain that on a cold day the bridges are very unpleasant on that account.—*Daily Advertiser.*

And Mr. Beecher, one of the bridge commissioners, says that if the fools that insisted on racing over the bridges had never been born or had been promptly drowned, or hanged, there would have been no need of signs, nor warnings; and decent citizens might be trotting their horses to their hearts' content. He says that a ton of coal walked over the bridges tries them more than three tons of fire engine crossing on a keen-jump. He says that he is glad the Lake street bridge sign of warning has blown down. He invites every one to trot until it is put up again. And further Mr. Beecher says: "When I am in a hurry I always trot the bridges, and what 're you going to do about it ? Ministers and fire engines are on life-and-death errands."

---

JOURNALISTIC ENTERPRISE.—The *Sunday Telegram*, of this city, has employed Ausburn Towner, Esq., to make a tour of the southern states and write a weekly letter to that journal, descriptive of the southern people and the many places made famous during the war. Mr. Towner started upon his journey on Christmas night and will stop at Washington, Fairfax C H., Richmond, Petersburg, Charleston, Savannah, Mobile, etc., bringing up at the New Orleans Exhibition.

This exhibits an enterprise and a desire to interest and please its patrons, on the part of the *Telegram*, not indulged in by journals outside of our largest cities. Ausburn Towner is well known as a very entertaining writer of fiction and travel, having been employed upon the New York *Herald*, the *Sun*, and other metropolitan journals, beside being the author of several successful novels. We predict a large increase in the *Telegram's* already enormous circulation by reason of Mr. Towner's contributions from the southern country.

FRITZ TRIES PUNCH.—It will be remembered that "Hellen's babies" demanded that a watch be opened that they might see the "wheels go wound." This exhibited a decided desire for scientific investigation on the part of the little ones that should not be ruthlessly discouraged. Fritz will be remembered as an agile chap, much after the "Hellen's-babies" sort, whose desire for investigation of matters and things not fully comprehended by him, induced him to investigate certain glasses found upon the dining-room table, one evening, after his father's guests had departed, and which his brother informed him was "punch." Fritz tasted thereof and found the flavor sweet and exceedingly agreeable to the palate, so that the feat of emptying all the glasses was easy of accomplishment. An hour afterward, Fritz was found under the table, curled up on the rug. When asked what he was doing there, replied, he was "watching the chairs go round."

---

"OUGHT TO BE MADE TO WORK.—There is a certain class of men arrested in this city for intoxication that would receive about the proper kind of treatment if they were sentenced to break stone eight hours a day for about thirty days. They hate work and they wont work if they can get out of it, and of what little they earn the largest share goes for whiskey. Al. Jordan is one of these men and this morning was sent to jail for twenty days. He leaves to the mercies of a cold world and the overseer of of the poor, a wife and four children, the oldest eight years of age and the youngest nine months. They live on North Elm street in a miserable condition, and this morning after Jordan was sentenced he besought the overseer of the poor, who was present, to send his family fifty cents worth of wood to keep them from freezing until he got out. It does not seem as though justice got her due, when such a shiftless, lazy fellow is supported in jail in comparative luxury to what he has been used to having, with nothing to do but smoke, play cards, eat and sleep, while his family is left to suffer through his misconduct."

So comments the *Gazette and Free Press*, of recent date, upon this exceedingly troublesome class of loafers. It reminded us of the treatment instituted for these criminals by the city of Indianapolis. Twenty five years ago, when we resided there, it was a daily occurrence to see twenty or more men with ball and chain fastened to one leg, and corraled by two armed guards, working upon the public highways. The city authorities thought it was no punishment to place these drunken loafers in jail, there to feed and warm them at the public expense, while they passed the time of their sentence in card-playing. So, they made them "pay their way" in the manner described. The scheme worked admirably then, (we do not know whether it is now in vogue or not) and we reccomend it to our efficient Mayor as an admirable method for "reforming" the drunken loafers: for, it was found there, that they very rarely appeared "on dress parade" the second time. Why not try the plan in Elmira?

---

FOREIGN MISSIONS.—Now and then, an earnest minded individual comes along, gains possession of the pulpits of city ministers and regales and interests the people with the woes, sufferings, lack of inteligence or religion of some unfortunate class of people located in a remote and difficult-to-approach country. These stories are always interesting, many times entertaining, and we doubt not, truthful. The people usually give liberally of their means to amelorate and improve the condition of the poor and unfortunate people of whose woes and miseries they have heard. While this promiscuous giving illustrates a generous impulse on the part of the sympathizing givers, is it just that they should send money to foreign missions, while, under their very eyes many of their own townspeople are suffering for the necessities of life? If they can afford to give to foreigners and to neighbors both, and do so, perhaps the act is excusable. But, we read in our daily newspapers of an aged negro having been found starved to death; of a widow and several children suffering for food, fuel and clothing; of an aged woman watching by the bed-side of her sick husband while she is unable to supply him with medical aid or much needed nourishment. These are narrations of occurences in our own midst, all appearing in our city papers within one week, while a neighboring village sends to our morning paper a story of poverty and suffering from which we make the following extract:

"The house in which this family of nine has been living was formerly an old fish house and is only eight by sixteen feet in dimensions. It is made of rough boards and has neither cellar nor garret. It stands on the bank of the lake where the cold wind has easy access through i's many crevices. Its interior presented a scene of destitution seldom seen in a civilized country. The only furniture was an old table with three legs, a chair, a dilapidated cook stove, and part of a bed. When the officers arrived the mother and two girls were out to find some wood and the little children sawing up the slats of the bed with which to build a fire. The floor was covered with filth and there was an entire absence of food in the house. How the family has lived is a mystery to everybody. The girls took the matter very cooly, evidently preferring a home in the jail to the miserable hut they were inhabiting. Straw, dirt and ashes in one corner of the room formed the bed on which the children slept. A jug without a handle, with a piece of wire attached was used for a teakettle; three broken plates and two cups composed all the dishes. During the past season it is said the children lived on bullfrogs that they caught and cooked in a fire made in the sand. The children's only apparel, the most of the time, was salt sacks, in the bottom of which a place was cut to put the head, and two holes in the sides for the arms. All of this destitution and abject poverty is within tho sound of our church bells where money is being raised weekly to send to the heathen who would blush at a sight like this."

Then, a friend in the city of Philadelphia sends us the following clipping from the *Times* of that city, showing that even in our large and wealthy cities, where it is supposed that abundant means are employed to prevent suffering and starving of unfortunate human beings; yet, men are found dying for lack of a friendly and able hand to relieve their distress. But. read this pathetic story, in which a dumb animal exhibits more affection for a human being than was shown by those of his own kind in a large city with thousands of men and women able to render assistance.

"The dismal howling of a dog attracted the attention of a policeman standing at the corner of Sixteenth and Jones streets at daybreak yesterday morning. The whines were continuous and were traced to an arch under the elevated road between Sixteenth and Seventeenth streets. He struck a light and found lying on a step the body of an old negro, guarded by a shaggy cur. The old man was insensible, but his brute companion received the officer with a clumsy welcome. Aid was summoned, and the old negro was carried to the Twentieth district police station, where he was recognized as James McFarlan, who had earned a living by picking rags and doing odd chores around the freight depots for years past. He is supposed to have been nearly eighty-five years old. He was still alive when taken to the station house, but died before the district physician arrived. His death was due to exposure.

While the body was being conveyed to the station house the dog walked meekly behind the procession and at the station watched the efforts of the officers to revive his master with great interest. Finally the animal stopped in his march around the stretcher on which the old man lay and sniffed anxiously about his head. Then followed a long-drawn, plaintive howl. The house sergeant leaned down and saw that the old negro was dead. The animal had detected this on the instant. Heretofore the dog had been perfectly docile and acquiesced in everything looking to the welfare of his master, but when two officers attempted to carry the stretcher into the corridor he struck an attitude and protested in vicious growls. So the body was allowed to remain where it was until the Coroner's undertaker arrived at about ten o'clock. Then the dog showed fight again, but was finally pacified into allowing the body to be carried in the wagon. He followed for some distance and then disappeared. At eleven o'clock last night the keeper of the Morgue was awakened by the wild howling and barking of a dog. He opened the door and found the cur that had been the old negro's companion for years."—*Phila. Times, Dec.* 25.

"Well, what of it? We cannot know of all such cases—if we did, of course, they would not be permitted to suffer and die," perhaps you reply. Precisely, that is just the question we desire to reply to, and will do so about as follows:

Instead of listening to and aiding in the finding of contributions of help to the poor and miserable beings of a foreign clime, give your money first to a *home* mission, possessing an officer whose duty it shall be to seek out the abiding places and the needs of just such cases as have been narrated above. Then, when no longer any suffering, cold, and hungry creatures exist in the city of Elmira, when the widow is not compelled to see her liltle ones shiver for lack of proper clothing or their frames grow emaciated for want of suitable food; when the faithful old wife is no longer compelled to watch helplessly by the bedside of her dying husband, without comfort or aid being offered by a living soul; when your charity has banished suffering and want from your own city, then, and not until then, give of your means, abundantly, to aid in the same good work on foreign shores, and may God bless you in the endeavor.

---

WHERE IS THE FAULT?—A young man recently applied to Rev. Thos. K. Beecher for aid in securing to himself some sort of employment to supply his pressing need whereby he might win food and clothing. "What can you do?" enquired Mr. Beecher. "I have no trade and am not skilled in *anything*," replied the boy. "Well, I will tell you what I think it best for you to do," said Mr. Beecher, after a moment's thought and having scrutinized the boy from head to foot. "You go to Stuart & Beech's store, watch your opportunity and when you think you can regain the door before being caught, seize the best roll of cloth there displayed—worth at least fifty dollars—and walk leisurely down the street with the package under your arm. You will soon be arrested. Go calmly with the officer and then send for me."

"Is that not rather unusual advice for a pastor to give to one of his flock?" we enquired. "And, furthermore, upon what ground do you render it?" To this query Mr. Beecher replied, in substance as follows He said the young man had not been taught any trade or vocation in life. Did not know how to use his hands or his head so that these members could be made available in securing to him sufficient income to pay the usual or necessary expenses of his existence. He could not afford to take lessons in book-keeping, telegraphy or other light employment, neither could he find any one who would receive him as an apprentice in learning a trade. There is no free-school where the means for gaining a livelihood are taught, save in the State Reformatory; where if he matriculates, he can be taught various trades, book-keeping, telegraphy, etc., while he is boarded and lodged in comfortable steam-heated apartments, without cost, until he graduates, fully capable to earn his own living. "I am sorry," continued Mr. Beecher, "that this boy must be advised to commit a crime against the law in order that he may gain that advantage which it would seem society owes him; but I know of no honest way by means of which that can be gained, and it does seem a pity that real rascals should profit by these very efficient free-schools, to the entire exclusion of honest but poor boys who *should* have the preference. "So, I gave him the advice I did, and, had he heeded it and acted upon it, I should have appeared before the courts, pleaded his case and advised, yes, begged the judge to send him to our excellent school—the State Reformatory."

Manifestly, there is something wrong here. But, who can say—and believe it—that Mr. Beecher's advice to that boy was not the best that could be given him? By no other means, save that of committing a crime—a crime only in the eye of the law—could that young man gain admission to Mr. Brockway's examplary and efficient school on the hill. Hundreds of real criminals receive the advantages to be gained by a free residence and schooling there. Why should not every boy—whose parents are too poor to give the instruction desired, and who manifest a real inclination for such tuition, be entitled to all these advantages? If they are entitled to our sympathy and help, how can we secure to them this excellent and timely aid? These are questions well worthy of the careful consideration of the authorities. What a blessing some thoughtful millionaire could confer upon the community in which he resides, in building and endowing just such a school for the benefit of those boys that have neither schooling nor handicraft!

---

THE CHOLERA.—All our medical journals come laden with warnings and advice about the cholera, which we are certified will certainly visit us upon the advent of hot weather. France and Italy have contributed thousands of lives to the ravages of this terribly fatal disease; and, since the germ or *microbe* is readily transferable to any foreign clime that offers it a suitable temperature and abundant nastiness in which to thrive and propagate itself, it becomes us to take timely warning and do all that within us lies to avert or at least mitigate its ravages.

Scientists, abundantly able to instruct us in sanitary matters, certify us that the cholera microbe flourishes best in such localities that harbor sewage, decomposing vegetable and animal substances, and then attack, by prefer ence, such individuals as do not pay strict attention to bathing, change of underwear, and in other ways do not keep their skins and bodies in a cleanly and wholesome condition. From such information it is plain that we had better do our share in frustrating the attempt of this miserable little microbe in gaining an abiding place on our shores.

Let us see to it that our cess-pools and vaults are not overflowing, spreading their breeding material over the surface of the ground. Use a plenty of quick lime in and about these re cepticles of filth. White-wash fences and buildings thereabout. Clean and white-wash all cellars, rake the yards and gutters—leaving nothing liable to decomposition above ground and sprinkle lime about plentifully. Be careful of diet, eating nothing that aforetime you have found disagreed with you. Partake of nothing likely to give you diarrhœa. Live simply, bathe frequently and fear nothing. Let not the stories of the approach of the disease frighten you into an attack of cholera. Remember, that when it does visit us, every belly-ache will probably receive the title of cholera. Be cool, collected, calm. Even should it attack your city, it is by no means certain that it will come to you, if you have taken the precautions we have pointed out to you.

Through the energy and capability of our health officer, Elmira has been pretty thoroughly policed. Should cholera come this way, not many cases would develop in our cleanly city, and those cases that will be found will nestle about the filthy localities where the health-officer's instructions have not been obeyed.

Therefore, we repeat, keep your premises, bodies and clothing clean. Select a wholesome diet, don't get frightened, and you will be in the best possible condition to resist the cholera microbe.

---

IN THE MATTER OF MALPRACTICE.—Occasionally, we learn through the public prints of actions that have been brought against surgeons for alleged malpractice, in which claims for large sums are laid for imaginable or apparent deformity which has occurred through non-union or malposition of fractured bones.

The instances are very rare in which any just cause for complaint against the surgeon is found, and we believe that such suits would be of less frequent occurence were a better understanding had, among the laity, of the duty and responsibility of the surgeon toward his patient.

To start with, it is unreasonable to suppose that the surgeon can, in *every* instance, restore a broken bone or adjust a dislocation, so that the parts are as good—if not better—than they were before the injury. There exist many examples of such injuries, where, despite the surgeon's skill and devoted care, deformity *will* result. As for instance, where the patient is violently thrown from a carriage, or falls from a great height, striking upon the palm of the hand or square upon the feet. Fractures of arm or leg, produced in this manner, are apt to be *impacted*, i. e., the ends of the broken bones are thrust together in such a manner as to materially shorten the limb, when no treatment or appliance that the surgeon can bring to bear upon the case, will restore it to its original form. Often, the ends of fractured bones are so jagged as to irritate the muscles in which they come in contact, forbidding the use of suitable dressings, because of the inability of the patient to endure the pain, unless under constant influence of anodynes or anæsthetics. By reason of some idiosyncrasy of the patient, the latter aids cannot be used, leaving the surgeon powerless to prevent deformity.

Many other contingancies arise in the treatment of fractures and dislocations which might be recited as militating against the very best efforts of the attendant to prevent deformity. The two instances given will suffice to show that they exist and that *sometimes* the surgeon is powerless to restore the parts to their former relations.

Then, too, if the patient would only use his own good judgement in considering his own case, before he rushes into law in an endeavor to obtain several thousand dollars from the surgeon for a result that could not be averted, he might conclude, after a few moments reflection, that the surgeon would not willingly neglect him, nor would he use other than his very best judgement and skill to restore him wholly from the injury sustained. Success in the treatment of such injuries is the surgeon's whole capital. It is upon his success that he thrives, while a single failure does him incalculable harm. It is unreasonable to suppose that he would not use every means within his power to prevent his patient from being a cripple. If he errs in judgement, even, and it seems plain that better results might have been obtained under different circumstances. still, the surgeon should not be considered as criminally at fault. Extend to him, at least, the same leniency that you would to a mechanic who did *his* best and failed in the result you desired.

---

ON THE ELECTION.—Dr. Blackham and Mr. Chandler have both written seasonable and non-partizan articles upon our method of electing a President of the United States. Their meditations have grown out of the manner and means used in the election just passed. Both agree—as we believe do a majority of the people—that our elections occur too frequently, that the President should be placed in office for at least eight or ten years, and thenceafter made ineligable for re-election. We call upon the readers of the BISTOURY to carefully consider this matter, talk it to their respective legislators and so aid in bringing about an amendment to the constitution that will enable us to accomplish this much needed reform.

